# α-Synuclein overexpression increases dopamine toxicity in BE(2)-M17 cells

**DOI:** 10.1186/1471-2202-11-41

**Published:** 2010-03-25

**Authors:** Marco Bisaglia, Elisa Greggio, Dragan Maric, David W Miller, Mark R Cookson, Luigi Bubacco

**Affiliations:** 1 Department of Biology, University of Padova, 35121 Padova, Italy; 2 Cell Biology and Gene Expression Section, Laboratory of Neurogenetics, National Institutes of Health, 20892 Bethesda, MD, USA; 3 Laboratory of Neurophysiology, National Institute of Neurological Disorders and Stroke, National Institutes of Health, 20892 Bethesda, MD, USA; 4 Current address: Department of Biology, University of Padova, Padova, Italy

## Abstract

**Background:**

Oxidative stress has been proposed to be involved in the pathogenesis of Parkinson's disease (PD). A plausible source of oxidative stress in nigral dopaminergic neurons is the redox reactions that specifically involve dopamine and produce various toxic molecules, i.e., free radicals and quinone species. α-Synuclein, a protein found in Lewy bodies characteristic of PD, is also thought to be involved in the pathogenesis of PD and point mutations and multiplications in the gene coding for α-synuclein have been found in familial forms of PD.

**Results:**

We used dopaminergic human neuroblastoma BE(2)-M17 cell lines stably transfected with WT or A30P mutant α-synuclein to characterize the effect of α-synuclein on dopamine toxicity. Cellular toxicity was analyzed by lactate dehydrogenase assay and by fluorescence-activated cell sorter analysis. Increased expression of either wild-type or mutant α-synuclein enhances the cellular toxicity induced by the accumulation of intracellular dopamine or DOPA.

**Conclusions:**

Our results suggest that an interplay between dopamine and α-synuclein can cause cell death in a neuron-like background. The data presented here are compatible with several models of cytotoxicity, including the formation of α-synuclein oligomers and impairment of the lysosomal degradation.

## Background

Parkinson's disease (PD) is a common neurodegenerative disorder characterized by symptoms that include resting tremor, slowness of movement, rigidity and postural imbalance. PD is a chronic and progressive disease caused by degeneration of several neuronal populations in the brain, most notably the dopaminergic neuromelanin-containing neurons of the *substantia nigra (SN) pars compacta *[1].

While the etiopathogenesis of idiopathic PD remains poorly understood, *post mortem *studies support the involvement of oxidative stress and the production of reactive oxygen species in neuronal damage [2,3]. Redox reactions that specifically involve dopamine (DA) are a possible source of oxidative stress accounting for the more pronounced degeneration of dopaminergic neurons in PD. A critical determinant of DA toxicity is the amount of DA present in the cytoplasm, outside of the acidic stabilizing environment of the synaptic vesicles where the neurotransmitter is normally confined. In the cytoplasm, DA can undergo both spontaneous or enzymatic-mediated oxidation and, as a consequence, generate superoxide anions, hydrogen peroxide and quinones [4], which can damage cellular components such as lipids, proteins and DNA [5-8].

Neuropathologically, PD is characterized by the presence of intracellular ubiquitinated inclusions, known as Lewy bodies, composed predominantly of fibrillar α-synuclein [9,10]. The discovery of three different missense mutations in the *SNCA *gene that codes for the α-synuclein protein, found in rare familial forms of PD indicates that α-synuclein is a likely causal factor in the pathogenesis of PD [11-13]. Further supporting this contention, multiplications of the *SNCA *gene are also causal for PD, suggesting that simply increasing the amount of protein is sufficient to trigger the disease [14]. Human α-synuclein is a small, 140-residue, natively unfolded protein abundantly expressed in neurons, where it is localized at presynaptic terminals [15-18]. The physiological role of α-synuclein is still poorly understood. Possible key functions of the protein could be the modulation of synaptic vesicle recycling, DA storage and release at nerve terminals [19-24]. In addition, α-synuclein could modulate intracellular DA handling through interactions with proteins that regulate DA synthesis and uptake, such as tyrosine hydroxylase [25], the aromatic amino acid decarboxylase [26] and plasma membrane dopamine transporter [27-29].

To explore the possibility of a synergistic effect between oxidative cellular conditions induced by the oxidation of DA and α-synuclein in PD, we investigated how wild-type (WT) α-synuclein or its A30P pathogenic mutant influences DA toxicity in a dopaminergic human neuroblastoma BE(2)-M17 cell model stably over-expressing α-synuclein.

## Methods

### Human Neuroblastoma Cell Lines

The production of stable cell lines overexpressing α-synuclein from parental BE(2)-M17 human dopaminergic neuroblastoma cells has been described elsewhere [30]. Cells were cultured in high-glucose DMEM (Life Technologies) supplemented with 10% FBS, 50 U/ml penicillin, 50 μg/ml streptomycin and 500 μg/ml G418. α-Synuclein expression was evaluated by Western blot analysis, using monoclonal anti-α-synuclein antibodies (BD Biosciences), after cell lysis and protein separation on SDS-PAGE. Films were analyzed with ImageJ (Rasband, W.S., ImageJ, U.S. National Institutes of Health, Bethesda, Maryland, USA, http://rsb.info.nih.gov/ij/) for densitometry quantification and protein levels were normalized with respect to β-actin.

### Lactate dehydrogenase (LDH) activity-based cytotoxicity assay

Cells were plated in 96 well plates at 1.5 × 10^4 ^cells per well in 100 μl of OptiMEM growth medium (Life Technologies) supplemented with DA or DOPA between 25 and 200 μM. Hydrogen peroxide generated by extracellular catecholamine oxidation was removed by adding 600 U/ml catalase (Sigma-Aldrich). After 24 hr of incubation, LDH activity measurements were performed using a commercially available assay (Roche Applied Science) according to the manufacturer's instructions. The ratio of LDH activity in the supernatant to the total LDH activity was taken as the percentage of cell death. For each experiment, eight wells per concentration were averaged and each experiment was performed in triplicate.

### Fluorescence-activated cell sorter (FACS) analysis

After 24 hr of incubation in the presence of 200 μM DA or DOPA, 10^6 ^cells/ml were labeled with Hoechst 33342 and propidium iodide (Vybrant Apoptosis Assay Kit #5; Molecular Probes, Invitrogen), according to manufacturer's specifications. Cells were analyzed using a dual-laser FACSVantage SE flow cytometer (Becton Dickinson, Mountain View, CA, USA). Propidium iodide was excited using a 488-nm laser light and the emission captured with a bandpass filter set at 613 ± 20 nm. Hoechst 33342 was excited using a 351-nm ultraviolet laser light and its emission captured with a bandpass filter set at 450 ± 20 nm. Cell Quest Acquisition and Analysis software (Becton Dickinson) was used to acquire and quantify the fluorescence signal intensities and to graph the data as bivariate dot density plots.

### Statistical analysis

Data were analyzed using GraphPad Prism 4 software and are expressed as the mean ± SEM. One-way ANOVA followed by Newman-Keuls's *post hoc *test was used to determine whether groups were statistically different. *P *values < 0.05 were considered significant.

## Results

**Figure 1:**
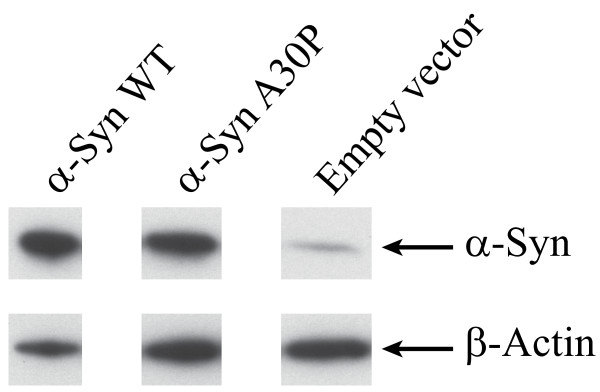
**Expression levels of α-synuclein in stably transfected M17 cell line**. Immunoblot of α-synuclein in cell lines overexpressing WT or A30P α-synuclein using a monoclonal antibody against α-synuclein (upper panel). The same immunoblot was then stripped and reprobed with an antibody for β-actin (lower panel). Quantitation of α-synuclein is expressed as a ratio between the major α-synuclein band and β-actin. Cells transfected with vector alone show moderate expression of α-synuclein compared to cells overexpressing WT or A30P α-synuclein.

**Figure 2:**
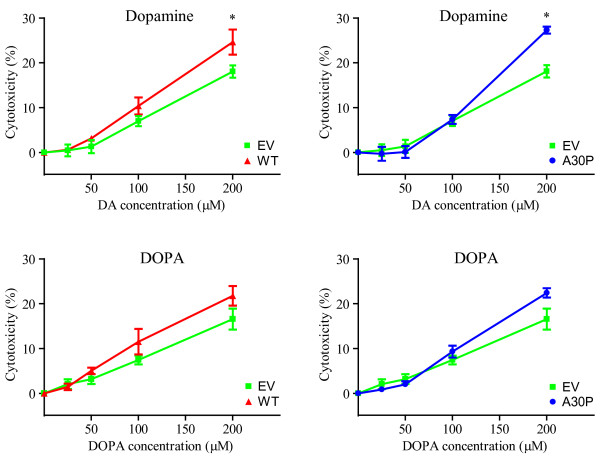
**α-Synuclein exacerbates DA or DOPA toxicity in M17 cells**. Overexpression of WT or A30P α-synuclein produces increased sensitivity to DA or DOPA. Cells were exposed to increasing concentration of catecholamines for 24 hr and cytotoxicity was subsequently estimated using the LDH assay (see Experimental Procedures). Cell lines included cells transfected with EV, WT or A30P α-synuclein. For each experiment, eight wells per concentration were used and each experiment was performed in triplicate. Values are the mean ± SEM. * p < 0.05

To study the relationship between α-synuclein and catecholamine toxicity, we used dopaminergic human neuroblastoma BE(2)-M17 cell lines stably transfected with WT or A30P mutant α-synuclein, as previously described [30]. The expression levels of WT and A30P α-synuclein were, respectively, about 8 and 6 times higher than cells transfected with the empty vector (Figure 1). Each cell line (empty vector control, WT or A30P α-synuclein) was treated with increasing concentrations (25, 50, 100 and 200 μM) of DA or DOPA, the cellular precursor of DA, and the levels of cellular toxicity were determined using an LDH assay. To limit the analysis only to the *intracellular *effects of DA or DOPA oxidation, we added 600 U/ml of catalase in the growth medium, as described for SH-SY5Y and PC12 cell types [31,32]. As expected, the presence of DA or DOPA in the growth medium induced cellular damage in all cell lines. The level of cell death was approximately 15% in the presence of the highest amount of catecholamines used in these experiments (200 μM) (Figure 2). Cells overexpressing WT or A30P α-synuclein showed an increased vulnerability to DA or DOPA-mediated toxicity (Figure 2), with a level of cellular death up to ~ 25% compared to controls. The toxicity induced by DA or DOPA was similar.

**Figure 3:**
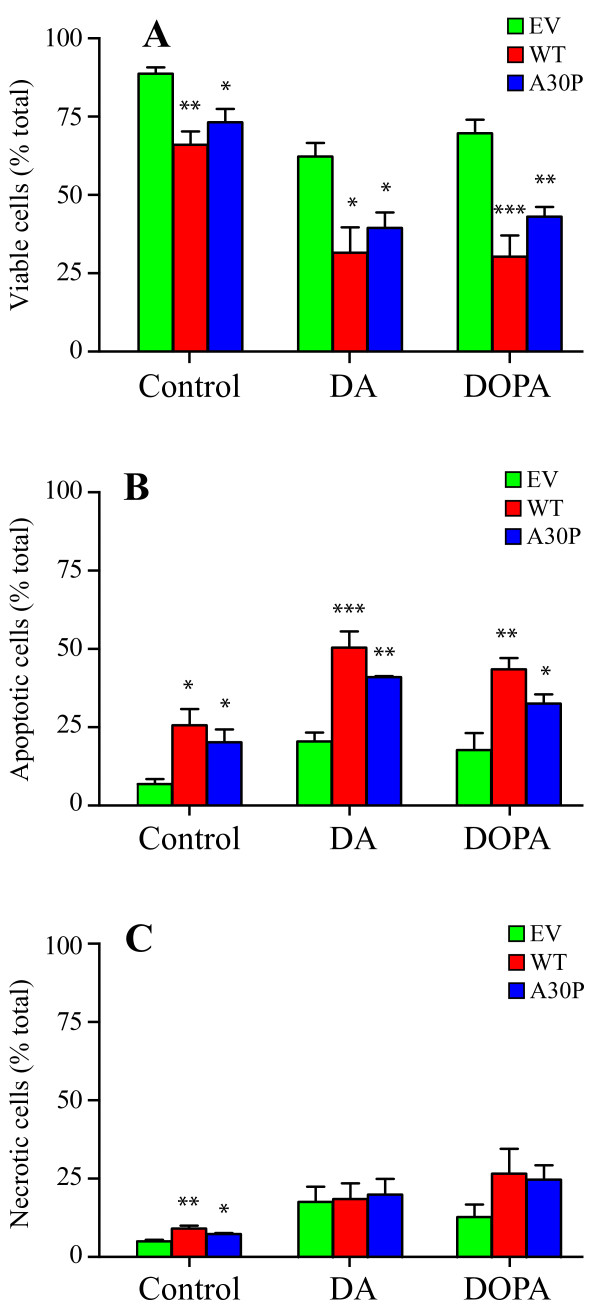
**DA and DOPA augment α-synuclein mediated apoptosis and necrosis**. Decreased cell viability (A) induced by DA or DOPA is accompanied by increased apoptosis (B) and necrosis (C). After 24 hr of incubation in the presence of 200 μM DA or DOPA, cells were labeled with Hoechst 33342 and propidium iodide and analyzed by FACS. For each experiment, 1.5-3 × 10^4 ^cells were analyzed. Values are the mean ± SEM (n = 4). * p < 0.05, ** p < 0.01, *** p < 0.001 relative to EV.

To confirm that overexpression of α-synuclein increases cell vulnerability to DA or DOPA, we performed analysis by fluorescence-activated cell sorting to discriminate between necrosis and apoptosis [33]. The analysis of 2 × 10^4 ^cells, obtained after 24 hr of incubation in the presence of 200 μM DA or DOPA, is shown in additional file 1. Figure 3 summarizes the results derived from 4 independent experiments. Addition of DA or DOPA to cells transfected with empty vector increased both apoptotic and necrotic events with a decrease in viability from 89% (control) to 62% (DA) and to 70% (DOPA). Although the cellular growth rate was similar both for empty vector and α-synuclein overexpressing cells, the presence of WT or A30P α-synuclein had cytotoxic effects with a decrease in viability from 89% to 66% (WT) and to 73% (A30P). The overexpression of WT or A30P α-synuclein variants also increased cellular susceptibility to DA or DOPA, especially for apoptotic cell death. The viability of cells overexpressing WT α-synuclein decreased from 89% to 32% (DA) and to 26% (DOPA), with and a change in viability of 57% and 63%, respectively. In the case of A30P overexpression, viability decreased from 89% to 39% (DA) and to 43% (DOPA), with a change in viability of 50% and 46%, respectively. Results obtained for each condition tested are summarized in Table 1. These data suggest that the cytotoxicity of α-synuclein and DA/DOPA are greater than the sum of the individual effects.

**Table 1:** Cell viability determined by FACS measurements

	EV	WT	A30P
**CNTR**	89% ± 2%	66% ± 4%	73% ± 4%
**DA**	62% ± 4%	32% ± 8%	39% ± 5%
**DOPA**	70% ± 4%	30% ± 7%	43% ± 3%

FACS measurements were carried out on cells transfected with empty vector (EV), WT or A30P α-synuclein variants, after 24 hr of incubation in the absence (CNTR) or in the presence of 200 μM DA or DOPA.

## Discussion

Oxidative stress and α-synuclein are considered two potential factors involved in the pathogenesis of PD. An important source of oxidative species is cytoplasmic DA which may contribute to the preferential neuronal death of catecholaminergic neurons observed in PD [34]. Previous studies have shown a direct correlation between α-synuclein and oxidized DA. In cell-free systems, oxidized DA can covalently modify α-synuclein and promote the stabilization of toxic protofibrils [35]. Moreover, degradation of α-synuclein through the chaperone-mediated autophagic pathway is impaired when the protein is modified by oxidized DA [36].

In the present work, we explored the combined effects of α-synuclein and DA in promoting cellular toxicity. The influence of α-synuclein on DA toxicity has been previously been mainly studied in transiently transfected cells or under inducible expression conditions [37-41]. In this study, we used stable transfected dopaminergic human neuroblastoma BE(2)-M17 cell lines expressing WT or A30P α-synuclein [30]. We found that the presence of either WT or mutant α-synuclein increase susceptibility to oxidative conditions induced by DA in this model.

Increased susceptibility to oxidative conditions upon WT or mutant α-synuclein overexpression is in agreement with studies using transient overexpresssion or inducible cell lines [37-41], but are in contrast with one previous study carried out using WT α-synuclein stably transfected SH-SY5Y cells [32]. The observed discrepancy is unlikely to be due to the fact that different cell lines were used as SY5Y cells have previously been shown to be susceptible to toxic effects of α-synuclein in a dopamine-dependent manner [38] but may be due to the choice of cytotoxicity assays, as catecholamines have been shown to interfere with MTT assay [32]. Because of this potential concern about interference of catecholamines with colorimetric assays, we used FACS analysis to confirm results seen in LDH release assays. This method allowed us to confirm that DA exposure and α-synuclein expression have additive toxic effects.

One possible interpretation for two observations described in this study, the toxicity measured in cells over expressing both WT and A30P α-synuclein relative to the control and the synergistic toxicity of α-synuclein and DA is that α-synuclein may have an effect on DA metabolism. It has previously been reported that overexpression of WT or mutant α-synuclein in PC12 cells [42] or in differentiated MESC2.10 cells [22], increases cytosolic catecholamine concentration. In contrast, the levels of cytosolic DA in adrenal chromaffin cells from mice over expressing human α-synuclein or from knock-out mice are unaltered compared to wild type animals [42,43], suggesting that the observation of the DA homeostasis effect by α-synuclein may be influenced by cell line. As we have not directly measured cytoplasmic DA concentrations in this cell line, further experiments are needed to define whether α-synuclein affects DA homeostasis in this context.

Another possible mechanism that may contribute to the combined toxic effects of α-synuclein and DA is the interaction between α-synuclein and the oxidation products of DA. In support of this hypothesis, we have shown *in vitro *that α-synuclein is able to incorporate radiolabeled DA [44]. The mechanism(s) underlying the potentially toxic effects of DA-modified α-synuclein are unclear. One leading hypothesis is that quinone adducts of α-synuclein may stabilize protofibrils [35]. Additionally, DA-modified α-synuclein blocks degradation of long-lived by chaperone mediated autophagy [36], an effect that can be seen with A30P α-synuclein without the incubation of the protein with DA [45]. Therefore, both impairment of the autophagy degradation and the formation of α-synuclein oligomers may both contribute to the observed cytotoxicity. Detergent-stable α-synuclein oligomers can isolated from DA-treated cells [41] and non-denaturing size fractionation also identifies non-covalent oligomers of α-synuclein in catecholaminergic cells [38]. However, whether these oligomers are specific to DA treatment, and therefore the same as the putative toxic species seen *in vitro*, is difficult to test as DA-modified α-synuclein has not yet been shown to be stable enough to be detected *in vivo*. Further work is therefore needed to establish whether the effects of DA and α-synuclein are mediated through direct interaction or independent pathways.

## Conclusions

In summary, we have shown that DA exposure increases the toxicity of the PD-related protein α-synuclein in dopaminergic neuroblastoma cell lines BE(2)-M17. These results support the concept that DA, likely through formation of cytosolic quinones, has synergistic toxic effects with α-synuclein. While additional studies are required to define the precise mechanism(s) by which the two stressors act in a cellular context, our results further highlight that an interaction between cytosolic DA and α-synuclein may underlie the susceptibility of SN neurons in PD.

## Abbreviations

DA: dopamine; FACS: fluorescence-activated cell sorter; LDH: lactate dehydrogenase; MTT: [3-(4,5-dimethylthiazol-2-yl)-2,5-diphenyl tetrazolium bromide]; PD: Parkinson's disease; WT: wild-type.

## Competing interests

The authors declare that they have no competing interests.

## Authors' contributions

MB, EG, DWM, MRC and LB designed experiments, MB, EG, DM and DWM carried out experiments, MB, EG, MRC and LB wrote the paper. All authors read and approved the manuscript.

## Supplementary Material

Additional file 1 **Figure S1. DA or DOPA induced toxicity analyzed by FACS**. After 24 hrs of incubation in the presence of 200 mM DA or DOPA, cells were labeled with Hoechst 33342 and propidium iodide. 2 × 104 cells were analyzed for each condition tested. The staining pattern resulting from simultaneous use of these dyes makes it possible to distinguish viable, apoptotic and necrotic cell populations. Cells transfected with the empty vector (A1-3) show moderate increase of apoptosis and necrosis after the incubation with either catecholamine. Apoptosis strongly increases in cell lines overexpressing both WT (B1-3) and A30P (C1-3) asyn variants. Necrosis is also increased after exposure to DA or DOPA, but to a lesser extent. Ap: apoptotic cells; N: necrotic cells; V: viable cells. Click here for file

## References

[B1] Hirsch E Graybiel AM Agid YA Melanized dopaminergic neurons are differentially susceptible to degeneration in Parkinson's disease Nature 1988 334 6180 345 348 10.1038/334345a0 2899295

[B2] Hirsch EC Does oxidative stress participate in nerve cell death in Parkinson's disease? Eur Neurol 1993 33 Suppl 1 52 59 10.1159/000118538 8375433

[B3] Jenner P Oxidative mechanisms in nigral cell death in Parkinson's disease Mov Disord 1998 13 Suppl 1 24 34 9613715

[B4] Graham DG Oxidative pathways for catecholamines in the genesis of neuromelanin and cytotoxic quinones Mol Pharmacol 1978 14 4 633 643 98706

[B5] Lotharius J Brundin P Pathogenesis of Parkinson's disease: dopamine, vesicles and alpha-synuclein Nat Rev Neurosci 2002 3 12 932 942 10.1038/nrn983 12461550

[B6] Ito S Kato T Fujita K Covalent binding of catechols to proteins through the sulphydryl group Biochem Pharmacol 1988 37 9 1707 1710 10.1016/0006-2952(88)90432-7 3132175

[B7] Hastings TG Lewis DA Zigmond MJ Role of oxidation in the neurotoxic effects of intrastriatal dopamine injections Proc Natl Acad Sci USA 1996 93 5 1956 1961 10.1073/pnas.93.5.1956 8700866 PMC39890

[B8] LaVoie MJ Hastings TG Dopamine quinone formation and protein modification associated with the striatal neurotoxicity of methamphetamine: evidence against a role for extracellular dopamine J Neurosci 1999 19 4 1484 1491 9952424 10.1523/JNEUROSCI.19-04-01484.1999 PMC6786023

[B9] Spillantini MG Schmidt ML Lee VM Trojanowski JQ Jakes R Goedert M Alpha-synuclein in Lewy bodies Nature 1997 388 6645 839 840 10.1038/42166 9278044

[B10] Spillantini MG Crowther RA Jakes R Hasegawa M Goedert M alpha-Synuclein in filamentous inclusions of Lewy bodies from Parkinson's disease and dementia with lewy bodies Proc Natl Acad Sci USA 1998 95 11 6469 6473 10.1073/pnas.95.11.6469 9600990 PMC27806

[B11] Polymeropoulos MH Lavedan C Leroy E Ide SE Dehejia A Dutra A Pike B Root H Rubenstein J Boyer R Mutation in the alpha-synuclein gene identified in families with Parkinson's disease Science 1997 276 5321 2045 2047 10.1126/science.276.5321.2045 9197268

[B12] Kruger R Kuhn W Muller T Woitalla D Graeber M Kosel S Przuntek H Epplen JT Schols L Riess O Ala30Pro mutation in the gene encoding alpha-synuclein in Parkinson's disease Nat Genet 1998 18 2 106 108 10.1038/ng0298-106 9462735

[B13] Zarranz JJ Alegre J Gomez-Esteban JC Lezcano E Ros R Ampuero I Vidal L Hoenicka J Rodriguez O Atares B The new mutation, E46K, of alpha-synuclein causes Parkinson and Lewy body dementia Ann Neurol 2004 55 2 164 173 10.1002/ana.10795 14755719

[B14] Singleton AB Farrer M Johnson J Singleton A Hague S Kachergus J Hulihan M Peuralinna T Dutra A Nussbaum R alpha-Synuclein locus triplication causes Parkinson's disease Science 2003 302 5646 841 10.1126/science.1090278 14593171

[B15] Nakajo S Shioda S Nakai Y Nakaya K Localization of phosphoneuroprotein 14 (PNP 14) and its mRNA expression in rat brain determined by immunocytochemistry and in situ hybridization Brain Res Mol Brain Res 1994 27 1 81 86 10.1016/0169-328X(94)90187-2 7877458

[B16] Iwai A Masliah E Yoshimoto M Ge N Flanagan L de Silva HA Kittel A Saitoh T The precursor protein of non-A beta component of Alzheimer's disease amyloid is a presynaptic protein of the central nervous system Neuron 1995 14 2 467 475 10.1016/0896-6273(95)90302-X 7857654

[B17] Weinreb PH Zhen W Poon AW Conway KA Lansbury PT Jr NACP, a protein implicated in Alzheimer's disease and learning, is natively unfolded Biochemistry 1996 35 43 13709 13715 10.1021/bi961799n 8901511

[B18] Bisaglia M Mammi S Bubacco L Structural insights on physiological functions and pathological effects of {alpha}-synuclein Faseb J 2008 23 2 329 40 10.1096/fj.08-119784 18948383

[B19] Abeliovich A Schmitz Y Farinas I Choi-Lundberg D Ho WH Castillo PE Shinsky N Verdugo JM Armanini M Ryan A Mice lacking alpha-synuclein display functional deficits in the nigrostriatal dopamine system Neuron 2000 25 1 239 252 10.1016/S0896-6273(00)80886-7 10707987

[B20] Murphy DD Rueter SM Trojanowski JQ Lee VM Synucleins are developmentally expressed, and alpha-synuclein regulates the size of the presynaptic vesicular pool in primary hippocampal neurons J Neurosci 2000 20 9 3214 3220 10777786 10.1523/JNEUROSCI.20-09-03214.2000 PMC6773130

[B21] Cabin DE Shimazu K Murphy D Cole NB Gottschalk W McIlwain KL Orrison B Chen A Ellis CE Paylor R Synaptic vesicle depletion correlates with attenuated synaptic responses to prolonged repetitive stimulation in mice lacking alpha-synuclein J Neurosci 2002 22 20 8797 8807 12388586 10.1523/JNEUROSCI.22-20-08797.2002 PMC6757677

[B22] Lotharius J Barg S Wiekop P Lundberg C Raymon HK Brundin P Effect of mutant alpha-synuclein on dopamine homeostasis in a new human mesencephalic cell line J Biol Chem 2002 277 41 38884 38894 10.1074/jbc.M205518200 12145295

[B23] Yavich L Tanila H Vepsalainen S Jakala P Role of alpha-synuclein in presynaptic dopamine recruitment J Neurosci 2004 24 49 11165 11170 10.1523/JNEUROSCI.2559-04.2004 15590933 PMC6730279

[B24] Larsen KE Schmitz Y Troyer MD Mosharov E Dietrich P Quazi AZ Savalle M Nemani V Chaudhry FA Edwards RH Alpha-synuclein overexpression in PC12 and chromaffin cells impairs catecholamine release by interfering with a late step in exocytosis J Neurosci 2006 26 46 11915 11922 10.1523/JNEUROSCI.3821-06.2006 17108165 PMC6674868

[B25] Perez RG Waymire JC Lin E Liu JJ Guo F Zigmond MJ A role for alpha-synuclein in the regulation of dopamine biosynthesis J Neurosci 2002 22 8 3090 3099 11943812 10.1523/JNEUROSCI.22-08-03090.2002 PMC6757524

[B26] Tehranian R Montoya SE Van Laar AD Hastings TG Perez RG Alpha-synuclein inhibits aromatic amino acid decarboxylase activity in dopaminergic cells J Neurochem 2006 99 4 1188 1196 10.1111/j.1471-4159.2006.04146.x 16981894

[B27] Lee FJ Liu F Pristupa ZB Niznik HB Direct binding and functional coupling of alpha-synuclein to the dopamine transporters accelerate dopamine-induced apoptosis Faseb J 2001 15 6 916 926 10.1096/fj.00-0334com 11292651

[B28] Wersinger C Prou D Vernier P Sidhu A Modulation of dopamine transporter function by alpha-synuclein is altered by impairment of cell adhesion and by induction of oxidative stress Faseb J 2003 17 14 2151 2153 12958153 10.1096/fj.03-0152fje

[B29] Fountaine TM Wade-Martins R RNA interference-mediated knockdown of alpha-synuclein protects human dopaminergic neuroblastoma cells from MPP(+) toxicity and reduces dopamine transport J Neurosci Res 2007 85 2 351 363 10.1002/jnr.21125 17131421

[B30] Ostrerova-Golts N Petrucelli L Hardy J Lee JM Farer M Wolozin B The A53T alpha-synuclein mutation increases iron-dependent aggregation and toxicity J Neurosci 2000 20 16 6048 6054 10934254 10.1523/JNEUROSCI.20-16-06048.2000 PMC6772599

[B31] Blum D Torch S Nissou MF Benabid AL Verna JM Extracellular toxicity of 6-hydroxydopamine on PC12 cells Neurosci Lett 2000 283 3 193 196 10.1016/S0304-3940(00)00948-4 10754220

[B32] Colapinto M Mila S Giraudo S Stefanazzi P Molteni M Rossetti C Bergamasco B Lopiano L Fasano M alpha-Synuclein protects SH-SY5Y cells from dopamine toxicity Biochem Biophys Res Commun 2006 349 4 1294 1300 10.1016/j.bbrc.2006.08.163 16978583

[B33] Hillion JA Takahashi K Maric D Ruetzler C Barker JL Hallenbeck JM Development of an ischemic tolerance model in a PC12 cell line J Cereb Blood Flow Metab 2005 25 2 154 162 10.1038/sj.jcbfm.9600003 15647748 PMC1378216

[B34] Chen L Ding Y Cagniard B Van Laar AD Mortimer A Chi W Hastings TG Kang UJ Zhuang X Unregulated cytosolic dopamine causes neurodegeneration associated with oxidative stress in mice J Neurosci 2008 28 2 425 433 10.1523/JNEUROSCI.3602-07.2008 18184785 PMC6670521

[B35] Conway KA Rochet JC Bieganski RM Lansbury PT Jr Kinetic stabilization of the alpha-synuclein protofibril by a dopamine-alpha-synuclein adduct Science 2001 294 5545 1346 1349 10.1126/science.1063522 11701929

[B36] Martinez-Vicente M Talloczy Z Kaushik S Massey AC Mazzulli J Mosharov EV Hodara R Fredenburg R Wu DC Follenzi A Dopamine-modified alpha-synuclein blocks chaperone-mediated autophagy J Clin Invest 2008 118 2 777 788 18172548 10.1172/JCI32806 PMC2157565

[B37] Tabrizi SJ Orth M Wilkinson JM Taanman JW Warner TT Cooper JM Schapira AH Expression of mutant alpha-synuclein causes increased susceptibility to dopamine toxicity Hum Mol Genet 2000 9 18 2683 2689 10.1093/hmg/9.18.2683 11063727

[B38] Xu J Kao SY Lee FJ Song W Jin LW Yankner BA Dopamine-dependent neurotoxicity of alpha-synuclein: a mechanism for selective neurodegeneration in Parkinson disease Nat Med 2002 8 6 600 606 10.1038/nm0602-600 12042811

[B39] Moussa CE Wersinger C Tomita Y Sidhu A Differential cytotoxicity of human wild type and mutant alpha-synuclein in human neuroblastoma SH-SY5Y cells in the presence of dopamine Biochemistry 2004 43 18 5539 5550 10.1021/bi036114f 15122920

[B40] Orth M Tabrizi SJ Tomlinson C Messmer K Korlipara LV Schapira AH Cooper JM G209A mutant alpha synuclein expression specifically enhances dopamine induced oxidative damage Neurochem Int 2004 45 5 669 676 10.1016/j.neuint.2004.03.029 15234109

[B41] Moussa CE Mahmoodian F Tomita Y Sidhu A Dopamine differentially induces aggregation of A53T mutant and wild type alpha-synuclein: insights into the protein chemistry of Parkinson's disease Biochem Biophys Res Commun 2008 365 4 833 839 10.1016/j.bbrc.2007.11.075 18039462

[B42] Mosharov EV Staal RG Bove J Prou D Hananiya A Markov D Poulsen N Larsen KE Moore CM Troyer MD Alpha-synuclein overexpression increases cytosolic catecholamine concentration J Neurosci 2006 26 36 9304 9311 10.1523/JNEUROSCI.0519-06.2006 16957086 PMC6674515

[B43] Mosharov EV Larsen KE Kanter E Phillips KA Wilson K Schmitz Y Krantz DE Kobayashi K Edwards RH Sulzer D Interplay between cytosolic dopamine, calcium, and alpha-synuclein causes selective death of substantia nigra neurons Neuron 2009 62 2 218 229 10.1016/j.neuron.2009.01.033 19409267 PMC2677560

[B44] Bisaglia M Mammi S Bubacco L Kinetic and structural analysis of the early oxidation products of dopamine: analysis of the interactions with alpha-synuclein J Biol Chem 2007 282 21 15597 15605 10.1074/jbc.M610893200 17395592

[B45] Cuervo AM Stefanis L Fredenburg R Lansbury PT Sulzer D Impaired degradation of mutant alpha-synuclein by chaperone-mediated autophagy Science 2004 305 5688 1292 1295 10.1126/science.1101738 15333840

