# Supplementary figures and images for: α-Synuclein overexpression increases dopamine toxicity in BE(2)-M17 cells
Source: BMC Neurosci. 2010 Mar 25;11:41. doi: 10.1186/1471-2202-11-41 (PMC2851596; doi:10.1186/1471-2202-11-41)

Control

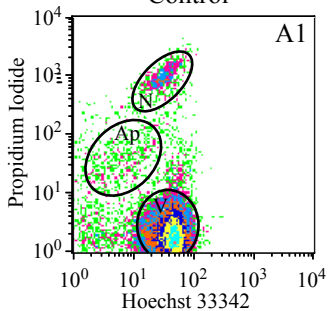

Dopamine

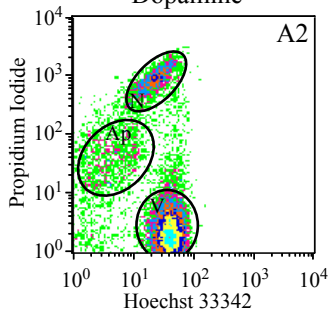

DOPA

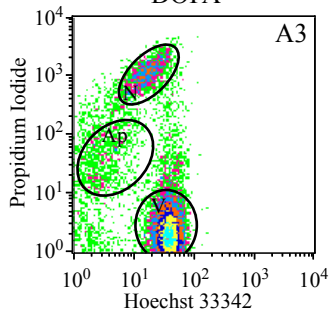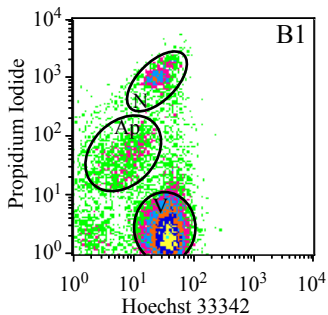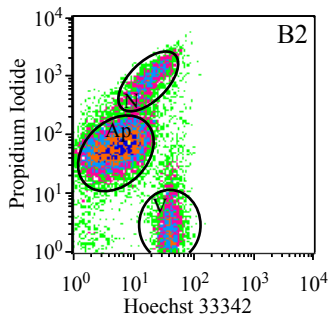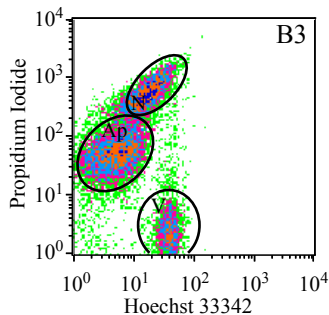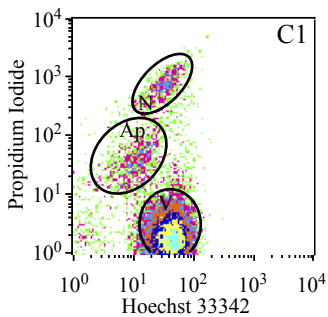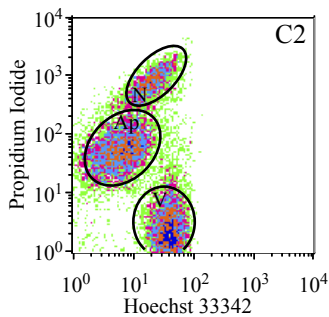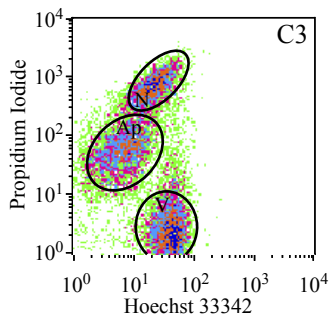

Supplement: Additional file 1 — Figure S1. DA or DOPA induced toxicity analyzed by FACS. After 24 hrs of incubation in the presence of 200 mM DA or DOPA, cells were labeled with Hoechst 33342 and propidium iodide. 2 × 10^4^ cells were analyzed for each condition tested. The staining pattern resulting from simultaneous use of these dyes makes it possible to distinguish viable, apoptotic and necrotic cell populations. Cells transfected with the empty vector (A1-3) show moderate increase of apoptosis and necrosis after the incubation with either catecholamine. Apoptosis strongly increases in cell lines overexpressing both WT (B1-3) and A30P (C1-3) asyn variants. Necrosis is also increased after exposure to DA or DOPA, but to a lesser extent. Ap: apoptotic cells; N: necrotic cells; V: viable cells. [file 1471-2202-11-41-S1.PDF]
